# A Profile of Novice and Senior Nurses’ Communication Patterns during the Transition to Practice Period: An Application of the Roter Interaction Analysis System

**DOI:** 10.3390/ijerph182010688

**Published:** 2021-10-12

**Authors:** Li-Fen Chao, Su-Er Guo, Xaviera Xiao, Yueh-Yun Luo, Jeng Wang

**Affiliations:** 1 Clinical Competency Center, Department of Nursing, College of Nursing, Chang Gung University of Science and Technology, Taoyuan City 33303, Taiwan; lfchao@mail.cgust.edu.tw (L.-F.C.); xavieraxiao@mail.cgust.edu.tw (X.X.); 2 Department of Emergency Medicine, Chang Gung Memorial Hospital Linkou Branch, Taoyuan City 33305, Taiwan; 3 Department of Nursing and Graduate Institute of Nursing, College of Nursing, Chang Gung University of Science and Technology, Chiayi County 613016, Taiwan; seguo@mail.cgust.edu.tw; 4 Chronic Diseases and Health Promotion Research Center, Chang Gung University of Science and Technology, Chiayi County 613016, Taiwan; 5 Division of Pulmonary and Critical Care Medicine, Chang Gung Memorial Hospital, Chiayi County 613016, Taiwan; 6 Department of Safety Health and Environmental Engineering, Ming Chi University of Technology, New Taipei 243303, Taiwan; 7 Department of Nursing, Ditmanson Medical Foundation Chia-Yi Christian Hospital, Chiayi City 600566, Taiwan; 8 Department of Nursing, Chang Gung Memorial Hospital, Linkuo Branch, Taoyuan City 33305, Taiwan

**Keywords:** communication, onboarding evaluations, interaction analysis, RIAS, transition period

## Abstract

Novice nurses’ successful transition to practice is impacted by their interactions with senior nurses. Ensuring that novice nurses are adequately supported during their transition to practice has wide-ranging and significant implications. The aim of this study is to explore the communication patterns between novice and senior nurses by applying an interaction analysis technique. Trimonthly onboarding evaluations between novice and senior nurses were recorded. The Roter Interaction Analysis System was adapted and deployed to identify communication patterns. In total, twenty-two interactions were analyzed. Senior nurses spoke more (64.5%). Task-focused exchange was predominant amongst senior (79.7%) and novice (59.5%) nurses. Senior nurses’ talk was concentrated in clusters of information-giving (45%) and advice or instructions (17.2%), while emotional expression (1.4%) and social talk (0.4%) were rare. Novice nurses’ talk was concentrated in clusters-information giving (57%) and positive talk (39.5%). The communication patterns between senior and novice nurses during the onboarding period indicate aspects of novice nurse transition that could be addressed, such as encouraging novice nurses to use these interactions to communicate more, or emphasizing the importance of social talk. These insights can be used to inform mentorship and preceptorship training to ensure that senior nurses are able to adequately support novice nurses through all parts of the transition to practice period.

## 1. Introduction

Communication between senior and novice nurses can have a significant impact on novice nurses’ transition to practice, their organizational commitment and their sense of psychological safety in the workplace [1,2,3,4]. During novice nurses’ onboarding, insufficient feedback and limited verbal engagement from senior nurses have been shown to have a negative impact on new nurses’ sense of confidence [5] and can exacerbate feelings of isolation [6]. By contrast, regular and constructive feedback from senior nurses has a valuable impact on novice nurses’ confidence and their professional development [7].

Ensuring that novice nurses are supported, both professionally and interpersonally, is important. Notably, novice nurses’ onboarding involves a significant amount of learning and adjustment. In addition to adapting to a new work environment and hospital information system, novice nurses must also take on a wholly new professional role. This involves engaging in new interpersonal dynamics and developing the clinical and professional skills needed to work independently and in groups. The struggle involved in adapting to these challenges can result in burnout and novice nurse turnover [8,9,10]. Indeed, the impacts of novice nurse burnout and turnover are experienced across global healthcare systems [11,12]. Novice nurse turnover exacerbates nursing shortages, leads to financial loss and lowers staff morale [8,13,14]. These circumstances highlight the importance of understanding the challenges faced by and increasing the support available for novice nurses during their transition to practice [15,16].

In order to tackle this problem, understanding the dynamics of senior and novice nurses’ communication is critical. In some studies, it has been pointed out that novice nurses benefit considerably from support from experienced nurses [17,18]. Notably, emotional support and social talk, which can facilitate workplace relationships, have been shown to have a positive impact on novice nurses’ commitment and willingness to stay in their role [19,20,21]. Interpersonal forms of workplace communication can help novice nurses as they negotiate the challenging aspects of their transition to practice [22]. By contrast, destructive forms of communication, which can include bullying and harassment, have been associated with increased turnover and intention to leave amongst novice nurses [23]. Even less explicitly harmful forms of poor communication, such as senior nurses failing to address workplace issues in a transparent and timely manner, have been identified as a factor that can lead to turnover [24].

Communication patterns between staff of differing rank and its impact on work culture have been examined in various work settings. One study examined the occurrence of workplace communication relating to tasks, relationships and safety to highlight the importance of supervisors approaching the topic of safety by emphasizing workers’ wellbeing [25]. Mikkelson [26] examined the components of relational communication in staff and supervisors’ talk and found that supervisors’ sense of dominance was negatively associated with job satisfaction, motivation and organizational commitment. Although research has increased our general understanding of workplace communication, there has been less research that has specifically explored communication patterns amongst nursing staff. Deconstructing the communication patterns that exist between senior and new staff can generate important insights into workplace dynamics and, by extension, highlight areas of senior nurse training that warrant greater attention. Chiefly, ensuring that senior nurses can facilitate open communication with novice nursing staff is integral to building positive working relationships, as well as supporting novice nurses who may encounter professional and personal challenges during their onboarding period.

To address this limitation within current research, our objective was to provide an exploratory investigation into novice and senior nurses’ communication dynamics. Specifically, our study aimed to identify the communication patterns between novice and senior nurses exhibited during trimonthly onboarding evaluations.

## 2. Materials and Methods

### 2.1. Study Design, Participants, and Setting

The study used an observational design. The Roter Interaction Analysis System (RIAS) was used to analyze audio recordings of senior and novice nurses’ interactions during novice nurses’ mandatory monthly onboarding evaluations. This study took place between July 2018 and November 2020 in the internal medicine department at a general hospital in a city in southern Taiwan. Convenience sampling was used to recruit novice and senior nurse participants employed at the hospital. In total, there were eleven new nurses who started between September 2018 to September 2019 and who were eligible to participate. The senior nurse participants were responsible for conducting monthly evaluations and had direct management responsibilities. This included chief nurse officers and nurse supervisors. The study was approved by the Institutional Review Board (CYCH-IRB2018053) of the hospital from which the participants were recruited. All the participants were provided with verbal and written information about the study and were required to give written consent to participate. The participants could withdraw at any time during the study. No identifying information was recorded.

### 2.2. Roter Interaction Analysis System

The Roter Interaction Analysis System (RIAS), created by Dr. Debra Roter and Susan Larson [27], includes software and a coding approach that facilitates an extensive breakdown of verbal interactions. This makes RIAS an especially unique tool, as the dynamics of an interaction can be examined through a quantitative lens [28]. While it is typically used to examine provider-patient interactions, the system can be adapted (through, for instance, the modification of the predetermined categories) to analyze interactions between different subjects (i.e., provider-provider). RIAS analyzes interactions by deploying software that allows the coder to apply preset communication categories, as well as coder-rated affect scores, directly on to the audio/audiovisual recording. All preset communication categories are considered either as socio-emotional exchange or as task-focused exchange. These categories can be further organized into communication clusters. For instance, categories such as disapproval-direct and criticism: general are placed in the cluster of negative talk. The categories are used to code all utterances (i.e., communication units) within an interaction. The global affect ratings require that the coder apply scores based on the role affects (e.g., anxiety, distress and respectfulness) play within the interaction [29]. These scores seek to understand the overall mood throughout the interaction. This is notable, as affect is a key component of verbal interaction that does not tend to receive sufficient attention within conventional qualitative analysis approaches [27]. The software is only available upon completion of a training course, wherein the learner is taught all the components of the coding process. The process of securing the software and the mandatory training ensure a degree of quality control.

### 2.3. RIAS Procedure

In line with the demands of RIAS, the study included two certified RIAS coders who had received relevant RIAS training and used RIAS in previous studies. At the outset, these RIAS practitioners (SH, JW) were assembled to adapt phenomenon-specific coding categories based on the interview context (i.e., the novice nursing staff evaluations) (see Table 1). As such, the categories developed by the research team enabled a degree of specificity and clarity. Indeed, the value of phenomenon-specific coding categories has been demonstrated in previous studies [30]. Following the development of context-specific categories, the coders practiced coding for two hours. During this time, the coders made sure to reach agreement on different aspects of the coding process to ensure consistency during the formal coding process.

### 2.4. Data Collection

The one-on-one mandatory evaluations took place in a private meeting room at the end of the first, second and third months of the new staff onboarding period. There were no time constraints imposed. These evaluations were intended to provide an opportunity for novice and senior nurses to discuss any issues that occurred and the overall onboarding process. All the interactions were recorded. In addition to the interaction data, the study also collected demographic and job characteristic data.

### 2.5. Data Analysis

There were two certified RIAS coders (JW, SH) who were responsible for coding all the data independently. In terms of the trustworthiness of the coding process, it is notable that the RIAS coding procedure has a built-in systematized approach. Whenever there is a degree of confusion regarding which category to apply, due to ambiguity in speech, the coder must refer to the preceding communication unit for clarification. The study found an average inter-rater reliability (kappa. 9) between the two coders on the total number of utterances and each of the categories contained therein. After each interaction was coded, the research team discussed discrepancies in coding until agreement on the category placement was achieved. Finally, descriptive statistics were used to analyze demographic and job characteristic data.

## 3. Results

### 3.1. Participant Characteristics

A total of seven senior nurses and all of the 11 eligible novice nurse staff members participated. Amongst the senior nurses, all of whom were female, there were five chief nurse officers and two nurse supervisors. Five senior nurses (71.4%) each had over 18 years of work experience in the nursing profession. Amongst the novice nurses, there were five males (45.5%). The novice nurses’ ages ranged between 20 to 27, with six participants (54.5%) between 21 and 23 years old. Eight novice nurses (72.7%) graduated from a bachelor program, while two of the novice nurses (18.2%) had prior work experience. For further demographic details, please refer to Table 2.

### 3.2. Trimonthly Evaluations

In the first month of evaluations, the longest session lasted 44 min; the average session was 22.2 (SD = 12.2) min. In the second month, the longest session lasted 42 min and the average session was 12.7 (SD = 14.5) min. In the third month, the longest session lasted 21.4 min and the average session was 11.7 (SD = 6.1) min (see Table 3).

### 3.3. The Roter Interaction Analysis System Findings

In total, 22 interactions were collected, comprising 10,903 utterances. The findings showed that senior nurses’ talk comprised the majority of the talk captured (64.5%). Task-focused exchange accounted for 79.7% of senior nurses’ talk and 59.5% of novice nurses’ talk. The findings demonstrated that the majority of communication from novice nurses was placed in the following clusters: information-giving (57.2%) and positive talk (39.5%). The findings showed that novice nurses’ communication had no instances of open-ended questions (0%) and no negative talk (0%); there were relatively few instances of close-ended questions (0.6%) and emotional responsiveness (0.8%) (see Table 4).

By contrast, senior nurses’ talk was placed into a greater variety of communication clusters. Similarly, information giving (45%) was the most prevalent form of communication from senior nurses, followed by advice or instructions (17.2%) and positive talk (12.4%). The least common types of communication engaged in were negative talk (*n* = 2, 0%) and social talk (*n* = 31, 0.4%), followed by emotional expression (1.4%), orientation (1%) and open-ended questions (3.4%) (see Table 4). In Table 5, a complete breakdown of each senior nurse’s talk is offered. It can be seen that senior nurses followed a similar approach, with all nurses engaging in more task-oriented communication. With the exception of one senior nurse participant (nurse E), social talk generally comprised between 0–1% of senior nurses’ talk.

The global affect ratings for talk from the participants in our study were analyzed based on rank, month and gender (see Table 6). The affect scores for interest/attentiveness, friendliness/warmth, responsiveness/engagement, respectfulness and interactivity were 6 across the different groups, which is the highest score possible. Anger/irritation received the score of 1, the lowest score possible, throughout. When examining the global affect ratings for novice nurses’ talk in the first, second and third month, it is notable that the affect scores for anger/irritation, interest/attentiveness, friendliness/warmth, responsiveness/engagement, hurried/rush, respectfulness and interactivity were consistent. Notably, depression/sadness went down from 1.2 in the first month to 1 in the third month. Sympathetic/empathy went down from 5.9 to 5.7. Dominance/assertiveness went down from 5.8 in the first month to 5.7 in the third month. Finally, anxiety and nervousness went down from 1.8 in the first month to 1.16 in the third month. Notably, the global affect ratings differed between male and female novice nurses. Male novice nurses demonstrated higher anxiety/nervousness at 1.75, compared to female nurses’ scores of 1.33. Male novice nurses also demonstrated higher scores for hurried/rush, depression/sadness and emotional distress/upset. Finally, male novice nurses demonstrated lower scores for dominance/assertiveness and sympathetic/empathy (Table 6).

## 4. Discussion

Our study contributes to a wider body of research that seeks to better understand aspects of novice nurses’ transition to practice, particularly in terms of how senior nurses engage with novice nurses. Our study achieved this by systematically deconstructing the communication dynamics between novice and senior nurses during monthly onboarding evaluations. In terms of contributing to RIAS studies, to the best of our understanding, this study makes a unique contribution by adapting the RIAS categories to examine provider-provider interactions. Moreover, while there were two previous RIAS studies carried out in Taiwan [31,32], our study appears to be the only study carried out in Taiwan that examines interactions between nurses.

Our study was able to recruit all novice nurses who were eligible. A total of 22 interactions were collected. Other RIAS studies have also relied on comparable sample sizes [33,34,35]. Ritter [36] used RIAS to analyze videos of 21 interactions; half of these interactions lost some amount of data due to minor logistical issues at either the end or the start of the video. Boss [28] analyzed 19 family conferences between parents of patients in the neonatal intensive care unit and healthcare providers.

Within our findings, one of the most evident distinctions in novice and senior nurses’ talk pertained to *who* spoke more. In our study, senior nurses spoke more during the evaluations, as their talk accounted for 64.5% of the talk captured. One possible explanation for the disparity in verbal communication during these interactions could be due to generational differences between the senior nurses (Generation X) and novice nurses (Millennials and Generation Z). Millennials and Generation Z nurses may be more likely to rely on online resources and social media to seek out technical answers and professional advice [37,38]. Notably, within our findings, it was evident that novice nurses did not utilize evaluations to ask questions. It is also possible that novice nurses may experience a certain amount of hesitancy to communicate, due to power imbalances. Previous studies carried out in Taiwan have highlighted that Confucian values can have a detrimental impact on professional relationships and the quality of communication in clinical settings, particularly for younger nurses [39,40]. In addition to generational differences, it is also possible that the type of communication between senior and novice nurses impacted novice nurses’ ability to engage. Indeed, the majority of communication between novice and senior nurses was centered on task-focused exchange, which may have contributed to senior nurses using this time to impart knowledge.

In addition, the findings showed that open-ended questions (3.4%) from senior nurses were relatively few, which may have impacted novice nurses’ ability and willingness to speak. Research on nurse leadership styles highlights the value of incorporating open-ended questions when communicating with incoming and novice nurses, as doing so enables students and novice nurses to express their thought process [41]. Through elaboration and discussion, senior nurses can encourage novice nursing staff to develop their clinical judgement and critical thinking through talk [41,42]. Ensuring senior nurse staff, who are given the considerable responsibility of training novice nurses in a new clinical environment, understand how to facilitate rich and open communication is key [43]. Future research may seek to explore how open-ended questions might improve engagement and the quality of communication between novice nurses and senior nurses who are tasked with the responsibility of mentoring incoming nursing staff.

The findings also demonstrated that social talk between senior (0.4%) and novice (0.2%) nurses was a conversation category that received relatively little attention. Creating opportunities for social talk can be especially important in cultivating positive group cohesion and interpersonal professional relationships [44,45]. Previous studies have pointed to the value of senior nurses engaging novice nurses during their transition period, to provide relevant professional support and discuss issues novice nurses may encounter [15]. Indeed, it has been found that when young novice nurses receive support and interpersonal care, this can have long-term and positive impacts on working relationships [46]. Future research may examine what inhibits this form of communication between senior and novice nurses. By extension, workshops run with senior nurses might focus on highlighting the importance of engaging in social talk as a way to better understand whether novice nurses are adapting successfully to the clinical setting and its demands.

Our findings also highlighted that the global affect ratings for male novice nurses’ talk indicated certain challenges in their transition to practice. The global affect ratings demonstrated that this faction of novice nurses showed more anxiety/nervousness, hurried/rush, depression/sadness and emotional distress/upset in their talk. This finding adds weight to existing research that suggests that male novice nurses face hurdles during their transition to practice. Studies have found that male nurses report discrimination, stereotyping and social isolation based on their gender identity [47,48]. When compared to other countries, Taiwan reports an especially low percentage of male nurses, with only 3.5% of the entire national nurse workforce identifying as male [49]. Future research may seek to understand how to train Taiwanese nursing leaders to support incoming staff from underrepresented groups during their transition to practice.

Ultimately, there were certain limitations to the study. Our study included 22 interactions. A larger sample, as well as additional interactions, would have yielded important insights. Indeed, our sample size does impact the generalizability of our findings. Therefore, it is critical to recognize that our study serves to add context and can provide insight for future studies that seek to continue to explore the communication dynamics between novice and senior nurses. It is also noteworthy that a lack of resources was a factor that determined how much data could be coded. Notably, the RIAS coding process is very time-consuming; 15 min of dialogue can take up to one hour to code. In effect, there were not sufficient resources to code additional data. Finally, our study recruited participants from one site and from one specialty. Future research may benefit from collecting interactions from additional sites and from novice nurses working in different specialties. Indeed, being able to compare findings from different clinical settings and cultural contexts may provide important insights.

## 5. Conclusions

Our findings indicate that during onboarding evaluations, senior and novice nurses tend to emphasize task-focused topics, while other aspects of the overall transition to practice period are not prioritized to the same extent. Additionally, these interactions tend to consist primarily of senior nurses’ talk, while novice nurses tend not to use these interactions to ask questions. Our research provides a starting point for future studies that seek to examine novice and senior nurses’ interactions. In addition, these findings can also be used to help shape the development of future initiatives that seek to train senior nurses to work alongside and support novice nurses. Future research may use these insights to examine how different communication approaches, such as encouraging novice nurses to ask more questions, may impact onboarding evaluations. As highlighted in this study, this is an important area of inquiry, given what is known about the challenges experienced by novice nurses as they enter the clinical context and, by extension, the role that senior nurses can play in helping novice nurses adapt to the new clinical environment. Understanding these interactions is a necessary step in determining what needs to be done to facilitate communication that supports novice nurses during their transition to practice.

## Figures and Tables

**Table 1:** Communication clusters and categories for senior and novice nurses’ talk.

#	Clusters	Categories in Roter Interaction Analysis System (RIAS)
1	Open ended questions	Independent/new employee orientation; handover; other nursing topics; psychosocial topics, including interpersonal interactions, prescriptions, future career discussions, non-nursing topics, patient condition; manager nurse mentorship
2	Closed-ended question	About university course; system operational problems; surroundings; future career discussions
3	Information giving	Independent/PGY plans; handover; other nursing topics; prescriptions; manager nurse mentorship; patient condition; environment; medical care process; colleague interaction; communication issues; system operational problems; future career discussions; medical condition; therapeutic regimen
4	Counsels or directs (*n*)	Behavior related to clinical condition; handover/therapeutic regimen information (*n*); patient condition; advice or instruction behavior relating to lifestyle and self-care; information/psychosocial feelings information; interpersonal interaction
5	Positive talk	Agreements; jokes and laughter; approval: direct
6	Negative talk	Disapproval-direct; criticism: general
7	Emotional expression (s)/responsiveness (*n*)	Concerns; reassurance; asks for reassurance (s); psychosocial-feelings; information (*n*); self-disclosure (*n*); empathy (s)
8	Facilitation	Asks for understanding; paraphrase and interpretation; asks for nurse opinion (n)
9	Social talk	Non-task; chit-chat; personal (e.g., “*Do you and your coworkers get together for dinner?*”)
10	Orientation (s)	Gives orientation; direct instructions (s) (e.g., “*Sign here, sign here*”)
11	Others	Information giving about other topics; open-ended question about other topics; closed-ended question about other topics; gives compliment-general; remediation; back-channel responses; legitimizing statements; empathy statements (*n*); bid for repetition; gives orientation; direct instructions (*n*); transition words; unintelligible utterances; information giving about psychosocial topics (*n*); asks for permission (*n*); asks for reassurance (*n*); partnership statement (s)
12	Job information giving composite	Give information-job; give information-care; give information-other; counsels-care/therapeutic
13	Job data gathering composite	Closed question-job; closed question-therapeutic; closed question-other; open question-job; open question-care; open question-other; bid for repetition
14	Psychosocial data gathering composite	Closed question-lifestyle; closed question-psychosocial; open question-lifestyle; open question-psychosocial
15	Psychosocial information giving composite	Give information-lifestyle; give information-psychosocial; counsels-lifestyle/psychosocial
16	Engagement composite	Ask for opinion; ask for permission; ask for reassurance; ask for understanding; back-channels; paraphrases
17	Procedural composite	Transition; give orientation; instruction (e.g., “*Actually my biggest goal with supervision is to let newcomers feel more at ease as they learn*”)
18	Emotional rapport-building composite	Empathy statement; legitimation statement; concern; worry; reassures; optimism; encourage; partnership statement; self-disclosure. Legitimation statement (e.g., “*The senior nurses they all slowly developed into their roles, it’s impossible that someone can just come in flying and be able to fly perfectly. Because everyone, when someone first starts to walk, aren’t they always stumbling? When a little kid is learning how to walk, they have to fall down a lot,”.* Self-disclosure (e.g., *“Afterwards we all feel, some doubt, why is it like this? One after another, there are many that get online and vent”).* Reassures; optimism; encourage (e.g *“All in all don’t worry, it seems like these leaders are all pretty friendly”*) Partnership (e.g., *“...at work, in terms of learning, is there anything you need help with?*”)

s: senior nurse; *n*: novice nurses.

**Table 2:** Participant characteristics *n* = 11 (novice nurse) and 7 (senior nurse).

Characteristics	Classification	Novice Nurse *n* (%)	Senior Nurse *n* (%)
Age	Mean	22.6 (SD = 2.30)	43.7 (SD = 3.15)
	Range	20–27	38–47
Gender	Male	3 (27.3)	
	Female	8 (72.7)	7 (100)
Education	Bachelor 5-year diploma	8 (72.7) 3 (27.3)	7 (100)
Work experience	No	9 (81.8)	
	Yes	2 (18.2)	
			15–24 (mean 18.9 yrs)
Title (senior)	Head Nurse Supervisor		5 (71.4) 2 (28.6)

**Table 3:** Trimonthly evaluations.

	1st Month	2nd Month	3rd Month
HN-NN interaction	9	--	7
SN-NN interaction	--	6	--
Duration (min)		Mean 16.3 (SD = 12.0)	
Mean (SD)	22.2 (12.2)	12.7 (14.5)	11.7 (6.1)
Max.	44	42	21.4
Min.	8.5	4.5	3.6

HN: Head nurse; NN: Novice nurse; SN: Supervisor nurse.

**Table 4:** Comparison of senior and novice nurse communication patterns: RIAS findings.

Pattern Clusters	Senior Nurse (Utterances *n* = 7034, 64.5%)		Novice Nurse (Utterances *n* = 3869, 35.5%)	
	Frequency	%	Frequency	%
Open-ended questions	240	3.4	0	0
Close-ended questions	689	9.8	25	0.6
Information giving	3166	45	2215	57.2
Advice or instructions (s)	1210	17.2	-	-
Positive talk	869	12.4	1528	39.5
Negative talk	2	0	0	0
Emotional expression (*n*)/responsiveness (s)	97	1.4	31	0.8
Social talk	31	0.4	9	0.2
Orientation (s)	71	1.0	-	-
Others	659	9.4	61	1.6
**Task-focus exchange**				
Gives orientation, instruction	4447	63.2	2215	57.2
Transition; check for understanding	227	3.2	63	1.6
Ask for opinions, understands	929	13.2	25	0.7
Subtotal	5603	79.7	2303	59.5
**Socioemotional exchange**				
Personal	31	0.4	9	0.2
Laugh	95	1.4	118	4.9
Concern, reassure	78	1.1	14	0.4
Approval, give compliment, agree	802	11.4	1355	35
Disagree, disapprove, criticisms	5	0	0	0
Empathy, legitimizing, partner	57	0.8	0	0
Back-channel, self-disclosure	363	5.2	0	0
Subtotal	1431	20.3	1566	40.5

S: senior nurse; N: novice nurse.

**Table 5:** Percentage of utterances of senior nurses (%).

Categories	% of Utterance from Senior Nurses						
	A	B	C	D	E	F	G
Open-ended questions	1	2	2	1	5	9	2
Close-ended questions	6	2	8	17	14	7	5
Information giving	29	35	37	22	37	41	28
Advice or instructions	28	19	5	2	15	24	12
Positive talk (agree, joke, laugh)	4	17	6	11	9	10	7
Responsiveness: self-disclosure, empathy	0	2	1	0	1	1	1
Social talk	0	0	0	1	14	1	0
Task focus exchange	90	69	83	62	78	83	78
Socioemotional exchange	10	31	17	38	22	17	22

**Table 6:** Global affect scores.

Affects	Senior	Nn Total	Nn 1st	Nn 2nd	Nn 3rd	Male	Female
Anger/irritation	1	1	1	1	1	1	1
Anxiety/nervousness	1	1.35	1.8	1.14	1.16	1.75	1.33
Dominance/assertiveness	6	5.7	5.8	5.57	5.7	5	5.83
Interest/attentiveness	6	6	6	6	6	6	6
Friendliness/warmth	6	6	6	6	6	6	6
Responsiveness/engagement	6	6	6	6	6	6	6
Sympathetic/empathetic	6	5.8	5.9	5.7	5.7	5.25	5.88
Hurried/rushed	1.1	1.1	1	1.14	1	1.25	1
Respectfulness	6	6	6	6	6	6	6
Interactivity	6	6	6	6	6	6	6
Depression/sadness	-	1.2	1.2	1.3	1	1.5	1.1
Emotional distress/upset	-	1.2	1.3	1.14	1	1.5	1.1

Nn: Novice nurse.

## Data Availability

The data that support the findings of this study are available from the corresponding author upon reasonable request.
